# Reply to López-Mañas et al.: Spatial population models of migrants should be underpinned by phenology, behavior, and ecology

**DOI:** 10.1073/pnas.2203349119

**Published:** 2022-05-05

**Authors:** Constanti Stefanescu, Gao Hu, Tom H. Oliver, Don R. Reynolds, Jason W. Chapman

**Affiliations:** ^a^Natural Sciences Museum of Granollers, E08402 Granollers, Spain;; ^b^Centre de Recerca Ecològica i Aplicacions Forestals, E08193 Bellaterra, Spain;; ^c^Department of Entomology, College of Plant Protection, Nanjing Agricultural University, Nanjing 210095, People’s Republic of China;; ^d^School of Biological Sciences, University of Reading, Reading RG6 6AS, United Kingdom;; ^e^Natural Resources Institute, University of Greenwich, Chatham ME4 4TB, United Kingdom;; ^f^Department of Computational and Analytical Sciences, Rothamsted Research, Harpenden AL5 2JQ, United Kingdom;; ^g^Centre for Ecology and Conservation, University of Exeter, Penryn TR10 9FE, United Kingdom;; ^h^Environment and Sustainability Institute, University of Exeter, Penryn TR10 9FE, United Kingdom

López-Mañas et al. (1) raise concerns about our recent paper (2) documenting environmental drivers of annual variation in the abundance of immigrant painted ladies (*Vanessa cardui*) reaching southern Europe each spring. We address their concerns, and further suggest that the rationale behind their critique is predicated upon unrealistic assumptions.

**Fig. 1.**
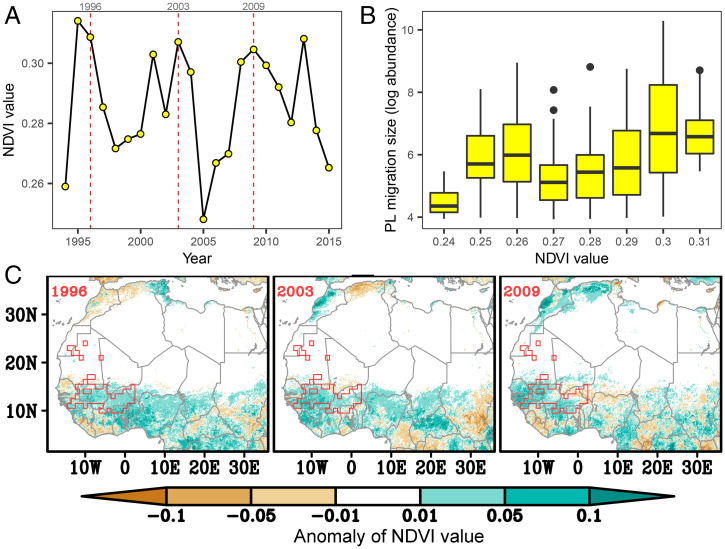
(*A*) January NDVI values in the western “kernel area” of the Savannah/Sahel during our study period (1994–2015); the red dashed lines indicate the 3 y with the largest spring arrivals in southern Europe, that is, the highest spring painted lady log-collated index values in NE Spain (2), within our study period. (*B*) The mean log abundance of painted ladies (PL) reaching NE Spain in each spring plotted against January NDVI in the west “kernel area”; there is a significant positive relationship (linear regression; *n* = 815, *r*^2^ = 0.073, *P* < 0.0001). (*C*). The January NDVI anomaly (compared to the overall mean between 1994 and 2015) across Africa for the three mass immigration years indicated in *A*; the outline of the west “kernel area” is delimited by the red polygons. Highly positive January NDVI anomalies can be seen in at least some parts of the west “kernel” in each of these years, indicating that conditions would have been more suitable for painted lady larval development than in more typical years.

Firstly, López-Mañas et al. (1) state that our identification of Savannah/Sahel “kernel areas” as important winter-breeding locations “challenges previous research in Africa” by these authors (3, 4). This is a somewhat surprising and contradictory viewpoint, as stable isotope analyses from the same group (5) pinpointed exactly the same location as our “kernel areas” as the source of late-winter arrivals in North Africa and Spain. López-Mañas et al. further surmise that high January–February normalized difference vegetation index (NDVI) values in the Savannah/Sahel in outbreak years correspond only to growth of woody plants, and that herbaceous larval hosts of *V. cardui* will be absent. Yet, a search of the Global Biodiversity Information Facility (https://www.gbif.org) for botanical records produces >1,000 occurrences of Asteraceae and ∼3,000 occurrences of Malvaceae [families including the principal *V. cardui* larval hosts (6)] within our “kernel areas” during January–February. We conclude that suitable larval hosts would be present at this key time, in at least some parts of the Savannah/Sahel, during years when NDVI was higher than average and large populations reached southern Europe in the spring (Fig. 1).

Secondly, López-Mañas et al. (1) assert that *V. cardui* has “low philopatry” with unpredictable spatiotemporal distribution patterns. Based upon their hypothesis, they contend that our population models (2) are flawed due to “predetermined spatiotemporal partition.” Their premise is erroneous, however, as the timing and general region of successive generations is, in fact, highly predictable and consistent across years. Irrefutable evidence for this come from 1) comprehensive long-term butterfly monitoring scheme data from multiple European countries (2); and 2) numerous published studies documenting dependable occupation of the same geographical regions, at the same seasons, across multiple years (3–5, 7, 8). Thus, we contend that constraining population models to realistic spatiotemporal distributions, as we do based upon our extensive knowledge of the migratory cycle (2, 9, 10), is more appropriate than ignoring feasible linkages between locations of successive generations. In particular, our analyses strongly suggest that positive winter NDVI anomalies in the Savannah/Sahel “kernel areas,” together with the assistance of southerly winds, are important in seeding or reinforcing the generation developing in the spring in the Maghreb and, ultimately, in explaining population levels recorded in Europe in spring and summer (Fig. 1).

We do not dispute additional populations of *V. cardui* migrants across Africa and the Middle East, although we do believe correlative analyses should be based on sound ecological theory and existing observations, such as of the massive spring emergence areas in Morocco (11)—the final leg of the African migration into Europe. Additional ground truthing through developing local monitoring capacity across Africa will provide further mechanistic understanding of the impressive transcontinental migration patterns of this butterfly.
